# The role of VASP in cGMP-mediated vascular smooth muscle relaxation

**DOI:** 10.1186/2050-6511-14-S1-P28

**Published:** 2013-08-29

**Authors:** Staffan Hildebrand, Katrin Zimmermann, Daniela Wenzel, Bernd K Fleischmann, Alexander Pfeifer

**Affiliations:** 1 Institute of Pharmacology and Toxicology, University of Bonn, Bonn, NRW, 53105, Germany; 2 NRW International Graduate School BIOTECH-PHARMA, Bonn, NRW, 53105, Germany; 3 Institute of Physiology I, University of Bonn, Bonn, NRW, 53105, Germany

## Background

Cyclic GMP (cGMP) is a major mediator of relaxation in the vascular system. cGMP is produced by the enzyme soluble Guanylyl Cyclase (sGC) in response to nitric oxide (NO) released from neighbouring endothelial cells. cGMP activates Protein Kinase G (PKG), which in turn mediates vascular relaxation through phosophorylation of various targets. One of the major substrates of PKG is the VAsodilator-Stimulated Phosphoprotein (VASP). The role of VASP in vascular smooth muscle relaxation is currently unknown. However, recent studies show that VASP-deficient brown adipocytes have an increased activity of the small GTPase Rac1 and elevated levels of sGC [1]. These data suggest a regulatory role for VASP in cGMP-mediated processes.

## Results

**Figure 1:**
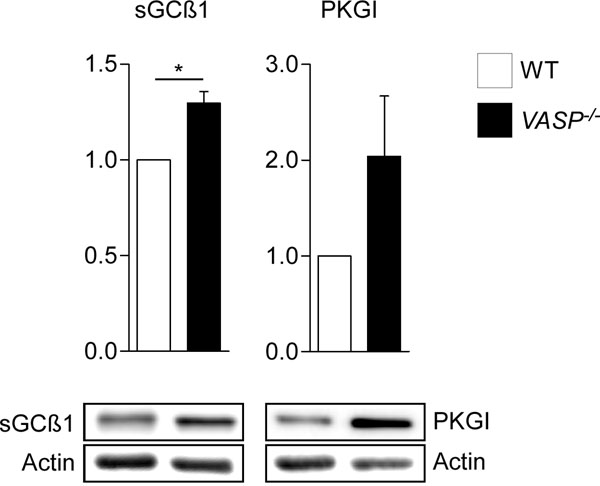
Expression of PKGI and the ß1-subunit of sGC in VASP-/- and wild-type murine aortas as determined by western blotting N=3.

Preliminary data acquired from the analysis of VASP-deficient (VASP-/-) mice provide evidence for the importance of VASP in the cGMP mediated relaxation pathway: VASP-/- aortas show higher levels of PKG and sGC compared to wild type (see Figure 1), and increased sensitivity to NO-induced relaxation. Additionally, cultured vascular smooth muscle cells (VSMCs) transduced with a constitutively active Rac1 mutant (RacL61) show elevated sGC and PKG expression as well as increased PKG activity. Cultured VSMCs from VASP-/- aortas also demonstrate decreased proliferation rates compared to wild-type cells in preliminary experiments.
